# Eye-Sight

**Published:** 1858-10

**Authors:** 


### EYE-SIGHT.

There are many persons who are greatly annoyed with weak eyes. This results, sometimes, from using the eyes in too great a glare of light; at others, from using them by artificial light, or in the "dusk of the evening;" that is, by twilight. To illustrate, in part: Our office has two large windows; twelve sets of slats cover them. If all the slats are open, our eyes begin to water in five minutes, and we commence a winking and a blinking, which has to be kept up all the time, to keep the sight clear. But if we close all the slats but one, and that an upper one, so as to throw the light down on the page, and from behind, we can write for hours, without inconvenience—or "specs." We conclude, therefore, that we have been using eleven times more light than was necessary. This simple fact may be of real practical use to multitudes. Too much light injures the eyes, as certainly as too little. We found this out to-day, after a variety of experiments within two months; thus, we are living to learn, with a view of learning to live—for a long time! The amount of relief afforded, in any particular case, can only be determined by experiment.

